# Correction: Application of Stochastic Labeling with Random-Sequence Barcodes for Simultaneous Quantification and Sequencing of Environmental 16S rRNA Genes

**DOI:** 10.1371/journal.pone.0173546

**Published:** 2017-03-03

**Authors:** Tatsuhiko Hoshino, Fumio Inagaki

There are errors in the fourth and fifth sentences of the Quantification by qSeq subsection of the Materials and Methods section. The correct sentences are: The SPE conditions consisted of initial denaturation at 98°C for 2 min, cooling to 55°C at 0.3°C/s, and elongation at 68°C for 10 min. Subsequently, excess primers were digested by addition of 4 μl of Exonuclease I (5 U/μl, TaKaRa Bio) and incubation at 37°C for 120 min, followed by inactivation of the enzyme by incubation at 80°C for 30 min.
